# Adverse Effects of Plant Food Supplements Self-Reported by Consumers in the PlantLIBRA Survey Involving Six European Countries

**DOI:** 10.1371/journal.pone.0150089

**Published:** 2016-02-29

**Authors:** Patrizia Restani, Chiara Di Lorenzo, Alicia Garcia-Alvarez, Mihaela Badea, Alessandro Ceschi, Bernadette Egan, Lorena Dima, Saskia Lüde, Franco M. Maggi, Angela Marculescu, Raimon Milà-Villarroel, Monique M. Raats, Lourdes Ribas-Barba, Liisa Uusitalo, Lluís Serra-Majem

**Affiliations:** 1 Dipartimento di Scienze Farmacologiche e Biomolecolari, Università degli Studi di Milano, Milano, Italy; 2 Fundación para la Investigacion Nutricional, Barcelona Science Park, University of Barcelona, Barcelona, Spain; 3 Transilvania University of Brasov, Brasov, Romania; 4 National Poisons Center, Tox Info Suisse, Associated Institute of the University of Zurich, Zurich, Switzerland; 5 Division of Clinical Pharmacology and Toxicology, Department of Internal Medicine, Ente Ospedaliero Cantonale, Lugano, Switzerland; 6 Department of Clinical Pharmacology and Toxicology, University Hospital Zurich, Zurich, Switzerland; 7 Food, Consumer Behaviour and Health Research Centre, University of Surrey, Guildford, Surrey, United Kingdom; 8 Finnish Food Safety Authority Evira, Helsinki, Finland; 9 Ciber Obn Fisiopatologıa de la Obesidad y la Nutrición, Instituto de Salud Carlos III, Madrid, Spain; 10 Institute of Biomedical and Health Research of Las Palmas, University of Las Palmas de Gran Canaria, Las Palmas de Gran Canaria, Spain; College of Tropical Agriculture and Human Resources, University of Hawaii, UNITED STATES

## Abstract

**Background:**

The use of food supplements containing botanicals is increasing in European markets. Although intended to maintain the health status, several cases of adverse effects to Plant Food Supplements (PFS) have been described.

**Objectives:**

To describe the self-reported adverse effects collected during the European PlantLIBRA PFS Consumer Survey 2011–2012, with a critical evaluation of the plausibility of the symptomatology reported using data from the literature and from the PlantLIBRA Poisons Centers' survey.

**Subjects/Setting:**

From the total sample of 2359 consumers involved in the consumers' survey, 82 subjects reported adverse effects due to a total of 87 PFS.

**Results:**

Cases were self-reported, therefore causality was not classified on the basis of clinical evidence, but by using the frequency/strength of adverse effects described in scientific papers: 52 out of 87 cases were defined as possible (59.8%) and 4 as probable (4.6%). Considering the most frequently cited botanicals, eight cases were due to *Valeriana officinalis* (garden valerian); seven to *Camellia sinensis* (tea); six to *Ginkgo biloba* (Maidenhair tree) and *Paullinia cupana* (guarana). Most adverse events related to the gastrointestinal tract, nervous and cardiovascular systems.

**Conclusions:**

Comparing the data from this study with those published in scientific papers and obtained by the PlantLIBRA Poisons Centers' survey, some important conclusions can be drawn: severe adverse effects to PFS are quite rare, although mild or moderate adverse symptoms can be present. Data reported in this paper can help health professionals (and in particular family doctors) to become aware of possible new problems associated with the increasing use of food supplements containing botanicals.

## Introduction

The European Union (EU) Directive on Food Supplements (2002/46/EC) defines food supplements (which include PFS): ‘‘…foodstuffs the purpose of which is to supplement the normal diet and which are concentrated sources of nutrients or other substances with a nutritional or physiological effect, alone or in combination, marketed in dose form, namely forms such as capsules, pastilles, tablets, pills and other similar forms, sachets of powder, ampoules of liquids, drop dispensing bottles and other similar forms of liquids and powders designed to be taken in measured small quantities”. Their market is growing significantly both in Europe and the USA [1]. Although there exists some overlap/confusion with traditional herbal medicinal products [2], plant food supplements cannot be sold as having any diagnostic, preventative or therapeutic properties; their role is only complementary to the diet.

The consumption of Plant Food Supplements (PFS) is usually estimated on the basis of market data, and mainly from import/export of raw ingredients, but since botanicals are used in both food and medicinal areas, the extrapolation to PFS is quite difficult [3]. Data on the use of dietary supplements reported by consumers are very limited and normally include only those products containing vitamins and minerals [4]; other available data come from studies relating to complementary/traditional medicine [5].

To provide new data on PFS usage patterns, a survey was performed with consumers of PFS in the framework of the European Project PlantLIBRA (n. 249159); it involved 2359 adults from Finland, Germany, Italy, Romania, Spain and UK. The main results of the PlantLIBRA PFS Consumer Survey (2011–2012) were published in 2014 [6]; the present paper deals with the adverse effects self-reported by the consumers participating in the survey.

Adverse effects to PFS have been reported by several authors; most of the studies were: a) case reports describing a specific acute event, or b) reviews of cases in a specific clinical area (cardiovascular, gastrointestinal, etc.) [7–8]. A critical limitation of the information reported to date in the scientific literature is a lack of assessment of causality; in other words, the strict association between the intake of a specific plant and the clinical event is rarely demonstrated by measuring biomarkers or by the de-challenge/re-challenge approach. On this basis, a systematic review of the data on adverse effects due to PFS/botanical ingredients, including misidentification and interactions of PFS/botanicals with pharmaceutical drugs or nutrients was undertaken [9]. Data were collected for 66 botanicals, which are common ingredients of PFS; all papers were classified for causality according to the WHO guidelines [10] and grouped as "certain, probable, possible and uncertain/unclassifiable events". Among the 492 papers selected, 402 (81.7%) dealt with adverse effects due to the botanical as such or in a PFS, and 89 (18.1%) described interactions with conventional drugs. Misidentification was confirmed in one case [9].

The aims of this paper are: 1) to identify the adverse effects reported by the European participants in the PlantLIBRA PFS Consumer Survey, and 2) to critically evaluate the plausibility of the symptomatology reported as being related to PFS.

## Materials and Methods

The survey was conducted in 6 European countries (Finland, Germany, Italy, Romania, Spain and the United Kingdom), and recruitment of participants occurred in 4 cities in each country. In this study, "Botanical" means raw material and derived preparations made from plants, algae, fungi or lichens (http://www.efsa.europa.eu/en/topics/topic/botanicals). The botanicals to be included in the survey were clearly defined at the outset; PFS were defined as the "foodstuffs the purpose of which is to supplement the normal diet and which are concentrated sources of botanical preparations that have nutritional or physiological effect, alone or in combination with vitamins, minerals and other substances which are not plant-based". Herbal remedies, other medicinal products based on botanicals, herbal teas or juices were excluded [6].

In order to obtain a sample of 400 consumers/each country, approximately 2000 individuals were screened per country (total number close to 2400) [6]. Eligible consumers completed a detailed questionnaire on PFS usage, providing product/plant names, dosage forms, frequency of use, reasons for use, adverse effects, places and patterns of purchase and information sources on products. Data on a maximum of five different PFS for each consumer was recorded; when PFS were more than 5, the inclusion was based on the frequency of use. Responders' sociodemographic data, including age, gender, level of education and employment status, as well as height, weight and health-related lifestyle information, were also collected. Further details on the survey have been reported previously [6]. The composition of each PFS was obtained from the label, if at disposal, or by searching the PFS ingredients in the website of producers.

Regarding the collection of data on adverse effects, the following two questions were included for each product:

1. Have you experienced any adverse effects while taking this product?
2. If yes, which one? (list of symptoms provided, with "other" as an option).

### Ethical aspects

Approval of the survey protocols was obtained from four ethics committees: the Bioethics Commission of the University of Barcelona, Spain; the Ethics Committee of the Università degli Studi di Milano, Italy; the Ethical Committee of the Faculty of Medicine—Transilvania University of Brasov, Romania; and the Coordinating Ethics Committee, Hospital District of Helsinki and Uusimaa, Finland.

Approval of the survey by these four ethics Committees required submitting all survey material to their members for evaluation. No ethical approval for the survey was needed in Germany and the United Kingdom. Furthermore, the ethical aspects were considered in the European Commission Consolidated Review Report dated 30^th^ September 2013 and evaluated as “ethical issues regarding the surveys have been handled appropriately”.

In all countries, informed consent was obtained from survey participants verbally after reading the survey information sheet. The data were collected anonymously on paper questionnaires and then transferred to an electronic database; all responders were assigned an ID number prior to data analyses.

### Statistical analysis

All data were entered into the statistical package SPSS for Windows v. 18 (IBM Corporation, Somers, NY, USA), which was used for analysis.

## Results and Discussion

A total of 11783 consumers (5799 males and 6004 females) were screened during the PlantLIBRA survey, of which 2359 were considered eligible and included in the study. The number of consumers per country and the percentage self-reporting adverse effects are listed in Table 1.

**Table 1:** Consumers included in the PlantLIBRA PFS Consumer Survey and self-reporting adverse effects.

Country		Number of consumers in the survey	Number of consumers reporting adverse effects	Percentage
Finland	Total number	401	23	5.7
	Males	193	10	5.2
	Females	208	13	6.3
Germany	Total number	398	22	5.5
	Males	197	13	6.6
	Females	201	9	4.5
Italy	Total number	378	5	1.3
	Males	187	2	1.1
	Females	191	3	1.6
Romania	Total number	400	7	1.8
	Males	199	4	2.0
	Females	201	3	1.5
Spain	Total number	402	24	6.0
	Males	174	9	5.2
	Females	228	15	6.6
United Kingdom	Total number	380	1	0.3
	Males	191	1	0.5
	Females	189	0	-
**Total**	**Total number**	**2359**	**82**	**3.5**
	**Males**	**1141**	**39**	**3.4**
	**Females**	**1218**	**43**	**3.5**

Considering the entire survey, the percentage of consumers, who reported adverse effects was approximately 3.5%. Differences were observed between countries; the number of consumers reporting adverse effects ranged between 5 and 6% of the total interviewed in three countries (Finland, Germany and Spain), while they were less numerous in Romania (2%), Italy (1%) and the United Kingdom (0.3%).

There were no significant differences based on sex or age groups (Table 1 and Table 2).

**Table 2:** Age of consumers included in the PlantLIBRA PFS Consumer Survey and of those reporting adverse effects.

Country	Consumers in the whole survey (m±SD)	Consumers reporting adverse effects (m±SD)
Finland	48.3±15.7	48.7±13.8
Germany	47.0±15.8	48.3±16.4
Italy	44.0±16.2	40.6±15.2
Romania	42.9±16.7	43.1±17.9
Spain	47.1±13.9	50.6±11.4
United Kingdom	48.9±14.2	35.0^a^
**Total**	**46.4±15.6**	**48.0±14.2**

m±SD = mean±Standard Deviation

^a^ no SD since only one consumer reported adverse effects

The data collected on adverse effects are presented in Table 3. Details of each of the 82 cases are recorded including:

- ID of the 82 consumers reporting adverse effects. ID 1–23 were from Finland, ID 24–45 from Germany, ID 46–69 from Spain, ID 70–74 from Italy, ID 75–81 from Romania, ID 82 from the United Kingdom. When a consumer reported adverse effects for two PFS, letters A and B follows the ID;
- Age and gender;
- The botanical/s contained in the PFS associated with the reported adverse effect. For a more precise identification, Latin names have been used but the corresponding common names are reported in Table 4 (see table note for scientific sources);
- The daily dose and the period of intake;
- The reason for use reported by the consumer, i.e. the physiological effect expected by the consumer;
- An assessment of the suitability of the botanical ingredient(s) present in the consumed PFS for the condition used, based on what the literature says about these ingredients. The main literature sources were the list of physiological effects published by the Italian Ministry of Health [11] and the EMA website [12]. In specific cases, other scientific papers were cited;
- The general health status of the consumer. These data allow a better assessment of adverse effect causality;
- Any reported simultaneous intake of conventional medicines and other food supplements; these data allow the assessment of possible interactions;
- The adverse effects reported by the consumers;
- A judgement as to the likelihood of causality, according to previous scientific citations and taking into consideration all available data.

**Table 3:** Adverse effects reported by PlantLIBRA PFS Consumer Survey participants.

ID	Age/Gender	Botanical/s^	Dose and period	Reasons for use	"Suitability"	Present or past main health problems	Conventional drugs + FS	Adverse effects	Causality
01	55/F	*Camellia sinensis*, *Panax ginseng*	1/d x 2w	Tonic	Yes [11]	Migraine	Antihistaminics, Corticosteroids, Ibuprofen, Roxithromycin + FO, PO, VM	Gastric problems	Possible worsening of gastric side effects of anti-inflammatory drugs
02	31/M	*Echinacea angustifolia*, *E*. *purpurea*	2/d x 2w	Immunity	Yes [11–12]	None	No drug + AA, VM	Gastric problems	Possible [43–44]
03	55/F	*Glycine max*	1/d x 6m	Menopause	Yes [11]	Allergy	Antiallergic drugs + Vitamin D	Gastric problems	Possible [45–46]
04	36/F	*Gossypium spp*., *Zingiber officinale*	2/d x 3m	Neuralgia	No	Allergy/asthma, joint/bone pain	Budesonide, Formoterol, Salbutamol + FO, V	Gastric problems	Possible [14; 47]
05	54/F	*Zingiber officinale*	1/d x 24d	Joints/bones	Yes [11]	HCHO	Simvastatin + PO, VM	Gastric problems	Possible [14; 47]
06	72/F	*Echinacea angustifolia*, *E*. *purpurea*,	3/d x 4d	Immunity	Yes [11–12]	HCHO	No drug + Vitamin D	Gastric problems	Possible [43–44]
07	57/F	*Echinacea angustifolia*, *E*. *purpurea*,	3/d x 3d	Immunity	Yes [11–12]	Cancer	No drug + FO, V	Tachycardia	Unlikely
08	44/F	*Achillea millefolium*, *Citrus aurantium*, *Crataegus spp*., *Daucus carota*, *Equisetum arvense*, *Foeniculum vulgare*, *Fucus vesiculosus* [alga], *Hibiscus rosa-sinensis*, *Ribes nigrum*, *Spinacia oleracea*, *Triticum spp*., *Urtica dioica*	2/w x 1m	Immunity, tonic	Unlikely	Migraine	No drug + VM	Gastric problems	Unassessable due to the presence of several ingredients
09	53/F	*Arctium lappa*, *Betula spp*., *Cichorium intybus*, *Cynara scolymus*, *Filipendula ulmaria*, *Foeniculum vulgare*	2/d x 1m	Detoxification	Yes [12]	None	No drug + AA, E, FO, PO, PE, VM	Increased diuresis	Possible due to the presence of diuretic ingredients [*Betula spp*., *Arctium lappa*] [48–49]
10	61/M	*Equisetum arvense*	3/d x 12m	Hair/skin	Yes [11]	None	No drug + FO, V	Hair loss/fragile nail	Possible due to decreased level of thiamine [50]
11	31/F	*Calendula officinalis*, *Citrus sinensis*, *Dunaliella salina* [alga], *Glycine max*, *Picea spp*.	1/d x 6m	Antioxidant	Limited evidence [13]	None	No drug + PO, VM	Gastric problems	Unassessable due to the presence of several ingredients
12	42/M	*Zingiber officinale*	1/d x 1m	Bodybuilding, tonic	Unlikely [14]	None	No drug + AA	Gastric problems	Possible [14; 47]
13	35/M	*Camellia sinensis*	2/d x 2m	Immunity, body weight, tonic, HCHO	Yes [11]	HCHO	No drug + Vitamin D, AA, FO	Insomnia and nausea	Possible for the content in caffeine [51]
14	39/M	*Olea europaea* (olive oil)	1/d x 9m	Immunity, hair/skin, tonic, mood, joints/bones, blood circulation	Yes [11]	Psoriasis	No drug + FO, PE, VM	Diarrhoea and nausea	Possible—high intake of olive oil could produce laxative effect [22]
15	72/F	*Oryza sativa + Monascus purpureus* [fungus]	1/d x 12m	HCHO	Yes [15–19]	Asthma, hypertension, cancer, depression, joint/bone pain	Acetylsalicylic acid, Amilodipine, Lisinopril, Pantoprazole + VM	Gastric problems	Possible [51–52] Possible worsening of gastric side effects of anti-inflammatory drugs
16	46/M	*Oryza sativa + Monascus purpureus* [fungus]	1/d x 12m	HCHO	Yes [15–19]	HCHO, hypertension	Drugs for hypertension + FO, PO, VM	Increased liver enzymes	Possible [52–53]
17A	70/M	*Oryza sativa + Monascus purpureus* [fungus]	1/d x 11m	HCHO	Yes [15–19]	HCHO, diabetes, hearth disease, allergy, depression	Acetylsalicylic acid, Enapril, Loratadine, Metoprolol, Mometasone + E, FO, PO, VM	Dry skin	Unlikely [52–53]
17B		*Plantago psyllium*, *Prunus africana*	1/d x 2m	Urinary tract	Yes [20]			Gastric problems	Possible [54] Possible worsening of gastric side effects of anti-inflammatory drugs
18	49/M	*Gossypium spp*., *Zingiber officinalis*	1/d x 2m	Sleeping, joints/bones	Unlikely	Muscle, joint/bone pain	Glucosamine + VM	Gastric problems	Possible [14, 47]
19	68/F	*Oryza sativa + Monascus purpureus* [fungus]	1/d x 12m	HCHO	Yes [15–19]	HCHO, hypertension, depression	Bisoprolol, Olanzapine, Thyroxin, Zopiclon + VM	Difficulty in swallowing	Possible [55]
20	46/M	*Oryza sativa + Monascus purpureus* [fungus]	1/d x 6m	HCHO	Yes [21–22]	HCHO, hypertension, diabetes	Metformin, Telmisartan + FO, PO, VM	Increased liver enzymes	Possible [52–53]
21	39/F	*Olea europaea*, *Melissa officinalis*	1/d x 2w	Immunity	Yes [21–22]	Migraine, allergy	Enoxaparin+ PO, VM	Allergic symptoms	Possible [56]
22	24/M	*Camellia sinensis*	1/d x 2m	Immunity, body weight, tonic, antioxidant	Yes [11–12]	None	No drug + E, FO, PO, VM	Nausea	Possible [57]
23	40/F	*Urtica dioica*	3/d x 2m	Body weight, immunity	Limited evidence [23–24]	None	No drug + AA, FO, PO, VM	"Easy" sweating	Possible [58]
24	48/F	*Arthrospira platensis* [alga]	1/d x 5m	Antioxidant, immunity	Yes [25–26]	Migraine	Analgesics + V	Insomnia	Uncertain
25	47/M	*Auricularia auricula-judae* [fungus], *Coffea arabica*, *Fallopia japonica/Polygonum cuspidatum*, *Ginkgo biloba*, *Panicum miliaceum*, *Polyporus umbellatus* [fungus], *Saccharomyces cerevisiae* [yeast], *Serenoa repens*, *Trigonella foenum-graecum*, *Ziziphus jujuba*	2/d x 12m	Hair/skin, energy	Yes [11, 27–28]	HCHO	None	Discomfort	Unassessable due to the presence of several ingredients
26	57/M	*Cucurbita maxima*, *Vaccinium macrocarpon*	2/d x 2m	Urinary tract	Yes [11]	HCHO, hypertension	Benazepril	Discomfort	Unassessable
27	45/M	*Saccharomyces cerevisiae* [yeast]	3/d x 3m	Hair/skin	Yes [29–30]	None	None	Skin problems	Unlikely [Allergy?]
28	42/M	*Asparagus officinalis*, *Cynara scolymus*, *Cichorium intybus (inulin)*, *Plantago psyllium*	1/d x 2w	Constipation	Yes [11]	None	No drug + M	Diarrhoea	Possible [59]
29	64/F	*Nigella sativa*	1/d x 1m	Immunity, HCHO	Yes [31–32]	None	No drug + M	Mild flatulence	Unlikely
30	42/F	*Cynara scolymus*	1/d x 6m	Digestion, HCHO	Yes [11–12]	HCHO	No drug + VM	Nausea	Possible [59]
31	62/F	*Glycine max*	3/d x 3m	Menopause	Yes [11]	Allergy	No drug + FO, M	Gastric problems	Possible [45–46]
32	31/M	*Matricaria recutita*, *Melissa officinalis*, *Valeriana officinalis*	3/d x 6m	Sleeping and mood problems	Yes [11–12]	Migraine, peptic ulcer, sleep disorders	None	Dizziness	Possible [60]
33	56/M	*Brassica oleracea*	2/d x 2m	Body weight	Unlikely	Hypertension, sleeping disorders, chronic bronchitis	None	Gastric problems	Unlikely
34	29/M	*Saccharomyces cerevisiae* [yeast]	2/d x 3m	Hair/skin	Yes [29–30]	None	None	Gastric problems, diarrhoea	Unlikely
35A	64/F	*Cynara scolymus*	1/d x 5d	Body weight, HCHO, digestion	Yes [12]	Hypertension, asthma, diabetes, joint/bone pain	Beclometason [spray], Formoterol [spray], Metformin, Thyroxin + VM, E (lactase)	Diarrhoea	Possible [22]
35B		*Camellia sinensis*, *Crataegus spp*., *Olea europaea* (olive oil), *Viscum album*	Unknown						Possible (olive oil)
36	27/F	*Olea europaea* (olive oil)	1/d x 5w	HCHO, body weight, digestion, blood circulation	Yes [11–12]	HCHO	No drug + FO, VM	Diarrhoea	Possible [22]
37	65/M	*Ginkgo biloba*	4/w x 12m	Memory	Yes [11–12]	HCHO	Iron supplementation, V	Insomnia	Possible [61]
38	66/F	*Ginkgo biloba*	5/w x 6m	Memory	Yes [11–12]	None	None	Constipation	Possible [62]
39	23/M	*Paullinia cupana*	1/d x 2m	Energy	Yes [11–12]	None	None	Diarrhoea	Unlikely
40	19/M	*Paullinia cupana*	2/w x 3w	Energy, urinary tract	Yes [11–12]	None	None	Constipation	Unlikely
41	71/M	*Cynara scolymus*	2/w x 4w	Antioxidant, immunity, digestion	Yes [11–12]	HCHO, hypertension	Metopolol, Ramipril	Gastric problems	Possible [59]
42A	31/M	*Peumus boldus*	2/w x 1w	Digestion	Yes [11–12]	None	None	Constipation	Unlikely
42B		*Linum usitatissimum*	2/w x 3w	Digestion	Yes [11–12]			Diarrhoea	Unlikely at the dose used
43	66/F	*Panax ginseng*	1/w x 6w	HCHO, relaxing, hair/skin	Yes [33–34]	HCHO, cataract	None	Constipation	Possible [63]
44	66/M	*Olea europaea*	5/w x 2w	Hair/skin	Unlikely	None	None	Gastric problems	Unlikely
45	41/F	*Oenothera biennis*	2/d X 3m	Immunity, hair/skin	Yes [11–12]	Hypertension, allergy	Antihypertensive drugs, Thyroxin	Mild eructation	Possible [64]
46	57/F	*Camellia sinensis*, *Paullinia cupana*	2/d x 2m	Body weight, digestion, energy/tonic	Yes [11–12, 35]	None	No drug	Insomnia	Probable due to the content in caffeine [35]
47	43/F	*Cassia angustifolia*, *Illicium verum*, *Raphanus sativus var*. *niger*, *Rhamnus purshiana*	1/d x 4w	Body weight, digestion	Yes [11]	Hypertension, migraine	No drug	Diarrhoea	Possible [65–66]
48	47/F	*Rhamnus purshiana*	1/d x 9m	Digestion	Yes [11]	Chronic neutropenia, glaucoma, vascular problems	Bimatoprost, Timolol	Gastric problems	Possible [66]
49	36M	*Valeriana officinalis*	4/w x 5m	Sleeping, relaxing, mood	Yes [11–12]	None	None	Insomnia	Unlikely but described [67]
50	61/M	*Punica granatum*	2/d x 12m	Prostate	Yes [36]	Cancer	None	Diarrhoea	Possible for high intake or previous intestinal disorders [68]
51	46/F	*Cassia angustifolia*, *Raphanus sativus var*. *niger*	1/d x 2m	Digestion	Yes [11]	None	None	Flatulence	Possible [65]
52	69/F	*Pimpinella anisum*	2/d x 12m	Digestion	Yes [11–12]	Hypertension, osteoporosis	Amlodipine	Diarrhoea	Uncertain (associated with allergic reaction)
53	61/F	*Valeriana officinalis*	2/d x 12m	Sleeping, relaxing, mood	Yes [11–12]	HCHO, heart disease, muscles, joint/bone pain, cataract	Alprazolam, Simvastatin	Constipation	Possible—abdominal cramps have been described [69]
54	72/F	*Valeriana officinalis*	1/d x 8m	Sleeping, memory, relaxing	Yes [11–12]	Cancer, joint/bone pain	None	Migraine	Possible [67]
55	36/F	*Panax ginseng*, *Paullinia cupana*	1/d x 3m	Energy/tonic	Yes [11–12]	None	Birth-control pill	Tachycardia	Probable [70]
56	39/M	*Passiflora incarnata*	1/d x 10m	Sleeping, relaxing	Yes [11–12]	Fatigue; insomnia	None	Insomnia	Unlikely
57	63/M	*Malus domestica*, *Citrus limon*	2/d x 4m	Constipation	Unlikely	HCHO, heart disease, hypertension	Quinapril/Hydrochlorthiazide, Diosmin/Esperidin [flavonoids]	Gastric problems	Unlikely
58	50/F	*Paullinia cupana*	1/d x 2w	Energy/tonic	Yes [11–12]	Hypertension, anxiety, depression	Fluoxetine	Tachycardia	Probable [70]
59	49/F	*Valeriana officinalis*	1/d x 3m	Relaxing	Yes [11–12]	HCHO; hypertension, migraine, allergy, anxiety	No drug + SI, VM	Flatulence	Possible—abdominal cramps have been described [69]
60	64/F	*Oenothera biennis*	3/d x 9m	Breast nodule	Yes [12]	Hypertension, allergy	Valsartan + AA, V, SI	Cystitis	Unlikely
61	60/F	*Harpagophytum procumbens*	3/d x 1m	Joints/bones	Yes [11–12]	Bone/joint pain, low back pain	None	Gastric problems	Possible [71]
62	42/M	*Allium sativum*	3/d x 2m	Immunity	Yes [37]	Asthma, renal problems	Amoxicillin/clavulinic acid	Allergic symptoms	Possible (quite rare)
63	49/M	*Taraxacum officinale*	3/d x 8m	Digestion, diuretic	Yes [11–12]	Liver disease	None	Diarrhoea	Unlikely
64	42/M	*Valeriana officinalis*	2/d x 4m	Sleeping, relaxing	Yes [11–12]	Liver disease, gallbladder inflammation	None	Insomnia	Unlikely but described [67]
65A	38/F	*Equisetum arvense*	2/d x 4m	Hair/skin, urinary tract	Yes [11–12]	Muscle and bone pain, migraine, ulcer, anxiety and depression, urinary problems	Trimethoprim, Sulfamethoxazol, Ibuprofen	Constipation	Possible—gastrointestinal complaints have been reported [72]
65B		*Taraxacum officinale*	1/d x 4m	Urinary tract	Yes [11–12]			Dizziness	Unlikely even though described for interaction with acetylsalicylic acid
66	46/F	*Lepidium meyenii*	2/d x 3m	Urinary tract [kidney stones]	Yes [38]	Allergy, kidney stones	Ibuprofen, Metamizole, Potassium citrate	Diarrhoea	Unlikely
67	54/M	*Echinacea angustifolia*	3/d x 1m	Flu cold	Yes [12]	HCHO, hypertension	Metformin, Olmesartan/Medoxomil	Increased glycemia	Unlikely
68	61/M	*Echinacea spp*	1/d x 2m	Sinusitis	Yes (cold) [12]	HCHO, anxiety and depression	Atorvastatin, Enalapril	Gastric problems	Possible [73]
69A	30/M	*Allium sativum*	1/d x 3m	Immunity, flu cold	Yes [37]	Allergy	None	Gastric problems	Possible [74]
69B		*Valeriana officinalis*	2/d x 12m	Sleeping	Yes [11–12]	Allergy	None	Migraine	Possible [67]
70	29/F	*Foeniculum vulgare*	3/d x 2m	Body weight, urinary tract	Yes [12]	Asthma, allergy	Beclometasone, Drospirenone/Ethinyl estradiol, Salbutamol	Difficult swallowing	Possible since reported in cases of allergy
71	35/M	*Paullinia cupana*	1d x 5m	Energy/tonic, mood	Yes [11–12]	Heart disease	None	Dizziness	Possible [75]
72	52/M	*Aloe barbadensis*, *Harpagophytum procumbens*	2d x 4w	Joints/bones	Yes [11–12]	Muscle, bone/joint pain	None	Unspecified	Unassessable
73	26/F	*Panax ginseng*	1d x 2w	Energy/tonic	Yes [11–12]	None	No drug + Inositol, folic acid	Tachycardia	Possible [76]
74	61/F	*Cyamopsis tetragonoloba*	20/m x ?	Body weight, energy/tonic	Unlikely [39–40]	Diabetes	None	Nausea	Possible [40]
75	69/M	*Ginkgo biloba*	2/d x ?	Joints/bones, blood circulation	Yes [11]	Diabetes, heart disease, hypertension, liver disease, stroke, gallbladder inflammation/stones	Acenocumarole, Captopril, Trimetazidine	Insomnia	Possible [77]
76	21/F	*Ginkgo biloba*	1/d x ?	Memory	Yes [11]	None	No drug + Polyphenols	Dizziness	Possible [78]
77	19/M	*Ginkgo biloba*	2/d x 14d	Memory	Yes [11]	Hypertension	Captopril	Insomnia	Possible [77]
78	41/F	*Arthrospira platensis* [alga], *Hippophae rhamnoides*	1/d x ?	Immunity, energy/tonic	Yes [11]	Anemia, arrhythmia	None	Gastric problems, nausea	Unlikely
79	50/M	*Camellia sinensis*	1/d x 2w	Immunity	Yes [41]	HCHO, diabetes, migraine	None	Diarrhoea, gastric problems (nausea)	Unlikely
80	49/M	*Camellia sinensis*	1/d x 2w	Immunity	Yes [41]	Migraine, ulcer	None	Diarrhoea, gastric problems (nausea)	Unlikely
81	53/F	*Betula spp*., *Equisetum arvense*, *Juniperus communis*, *Pimpinella anisum*, *Vaccinium vitis-idaea*	3/d x 20d	Urinary tract	Yes [11]	HCHO, asthma, diabetes, heart disease, hypertension, liver disease, chronic bronchitis, cataract, osteoporosis, allergy, cancer, Basedow disease	Enalapril, Metformin, Nicergoline, Simvastatin	Gastric problems	Unassessable due to the presence of several ingredients
82	35/M	*Aloe vera*	3/w x 12m	Joints/bones	Yes [42]	None	None	Diarrhoea	Probable [laxative effect]

^ according to: for plants US Department of Agriculture (plants.usda.gov); for algae www.algaebase.org; for fungi www.indexfungorum.org

? unknown

AA= Supplement containing amino acids; FO= Fish Oil; E= Enzymes; HCHO= Hypercholesterolemia; M= Supplement containing minerals; PE= Prebiotics; PO= Probiotics; SI= Soy isoflavones; V= Supplement containing vitamins; VM= Supplement containing vitamins and minerals; d= day; m= month; w= week

**Table 4:** Botanical ingredients contained in PFS with reported adverse effects.

*Latin name*^	Common name^	Number of counts										
		Total for country							TOTAL	1 ING^a^	2–3 ING	≥ 4 ING
		FI	D	IT		RO	SP	UK				
*Valeriana officinalis*	Garden valerian		1				7		**8**	7	1	
*Camellia sinensis*	Tea	3	1			2	1		**7**	4	2	1
*Ginkgo biloba*	Maidenhair tree		3			3			**6**	5		1
*Paullinia cupana*	Guarana		2		1		3		**6**	4	2	
*Cynara scolymus*	Globe artichoke	1	4						**5**	3		2
*Echinacea angustifolia/purpurea*	Black Samson Echinacea/ Eastern purple coneflower	3					2		**5**	5		
*Olea europaea*	Olive	2	3						**5**	3	1	1
*Oryza sativa + Monascus purpureus*	Red rice	5							**5**	5		
*Panax ginseng*	Chinese ginseng	2	1		1		1		**5**	2	3	
*Equisetum arvense*	Field horsetail	2				1	1		**4**	2		2
*Allium sativum*	Cultivated garlic						3		**3**	3		
*Foeniculum vulgare*	Sweet fennel	2			1				**3**	1		2
*Glycine max*	Soybean	2	1						**3**	2		1
*Saccharomyces cerevisiae*	Yeast		3						**3**	2		1
*Aloe barbadensis/vera*	Barbados aloe				1			1	**2**	1	1	
*Arthrospira platensis*	Spirulina		1			1			**2**	1	1	
*Betula spp*.	Birch	1				1			**2**			2
*Cassia angustifolia*	Alexandrian senna						2		**2**		1	1
*Citrus aurantium*	Sour orange	2							**2**			2
*Crataegus spp*.	Hawthorn	1	1						**2**			2
*Cichorium intybus*	Chicory	1	1						**2**			2
*Gossypium spp*.	Cotton	2							**2**		2	
*Harpagophytum procumbens*	Devil's claw				1		1		**2**	1	1	
*Melissa officinalis*	Common balm	1	1						**2**		2	
*Oenothera biennis*	Common evening primrose		1				1		**2**	2		
*Pimpinella anisum*	Anise burnet saxifrage					1	1		**2**	1		1
*Plantago psyllium*	Psyllium	1	1						**2**		1	1
*Raphanus sativus var*. *niger*	Spanish black radish						2		**2**		1	1
*Rhamnus purshiana*	Cascara buckthorn						2		**2**	1		1
*Taraxacum officinale*	Common dandelion						2		**2**	2		
*Urtica dioica*	Stinging nettle	2							**2**	1		1
*Zingiber officinale*	Garden ginger	2							**2**	1	1	
*Achillea millefolium*	Common yarrow	1							**1**			1
*Arctium lappa*	Greater burdock	1							**1**			1
*Asparagus officinalis*	Garden asparagus		1						**1**			1
*Auricularia auricula-judae*	Jew's ear		1						**1**			1
*Brassica oleracea*	Cabbage		1						**1**	1		
*Calendula officinalis*	Pot marigold	1							**1**			1
*Citrus limon*	Lemon						1		**1**		1	
*Coffea arabica*	Arabian coffee		1						**1**			1
*Cucurbita maxima*	Winter squash		1						**1**		1	
*Cyamopsis tetragonoloba*	Guar				1				**1**	1		
*Daucus carota*	Carrot	1							**1**			1
*Dunaliella salina*	"Green alga"*	1							**1**			1
*Fallopia japonica*	Japanese knotweed		1						**1**			1
*Filipendula ulmaria*	Queen of meadow	1							**1**			1
*Fucus vesiculosus*	Bladder wrack	1							**1**			1
*Hibiscus rosa-sinensis*	Shoeblackplant	1							**1**			1
*Hippophae rhamnoides*	Seaberry					1			**1**		1	
*Illicium verum*	Staranise tree						1		**1**			1
*Juniperus communis*	Common juniper					1			**1**			1
*Lepidium meyenii*	Maca						1		**1**	1		
*Linum usitatissimum*	Common flax		1						**1**	1		
*Malus domestica*	Apple						1		**1**		1	
*Matricaria recutita*	German chamomile		1						**1**		1	
*Nigella sativa*	Black cumin		1						**1**	1		
*Panicum miliaceum*	Proso millet		1						**1**			1
*Passiflora incarnata*	Purple passionflower						1		**1**	1		
*Picea spp*.	Spruce	1							**1**			1
*Peumus boldus*	Boldo		1						**1**	1		
*Polyporus umbellatus*	Umbrella polypore		1						**1**			1
*Prunus africana*	Red stinkwood	1							**1**		2	
*Punica granatum*	Pomegranate						1		**1**	1		
*Ribes nigrum*	European blackcurrant	1							**1**			1
*Serenoa repens*	Saw palmetto		1						**1**			1
*Spinacia oleracea*	Spinach	1							**1**			1
*Trigonella foenum-graecum*	Sicklefruit fenugreek		1						**1**			1
*Triticum spp*.	Wheat	1							**1**			1
*Vaccinium macrocarpon*	Cranberry		1						**1**		1	
*Vaccinum vitis idaea*	Cowberry/lingonberry					1			**1**			1
*Viscum album*	European mistletoe		1						**1**			1
*Ziziphus jujuba*	Common jujube		1						**1**			1
**Total counts**		48	42		6	12	35	1	**144**	66	28	50
**Percentage of the total**		33	29		4	8	24	0.7	100	45.8	19.4	34.7

^ according to: for plants US Department of Agriculture (plants.usda.gov); for algae www.algaebase.org; for fungi www.indexfungorum.org

* no common name

FI = Finland; D = Germany; IT = Italy; RO = Romania; SP = Spain; UK = Unided Kingdom

^a^ING = Ingredients (number of botanicals contained in the product associated with the adverse effect)

Considering the suitability of the botanical product used in relation to the physiological effect expected by the consumers, the choice was considered appropriate in 88% of cases. One case (ID 4) was considered non pertinent and nine (ID 8, 11, 12, 18, 23, 33, 44, 57, 74) judged as unlikely or with limited evidence. One consumer (ID 08) used a product containing 12 herbal ingredients, but only *Citrus aurantium* could claim to have tonic properties due to the presence of active amines. None of the herbal ingredients present have any reported immune activity [11–12].

Since all cases were self-reported, it was not possible to establish causality of adverse effects on the basis of clinical evidence. The scientific literature was used to assess the likelihood of the adverse effects being associated with the botanical used and 56 out of 87 (64%) cases were defined as possible (52) or probable (4) according to 1) the daily dose and period of intake, and 2) the frequency and strength of scientific evidence. The most significant references are reported in Table 3. The association was not confirmed for 28 cases. The interaction with conventional drugs was considered possible in three cases (ID 01, 15 and 17B). It is important to underline that comparing the list of conventional drugs used with the column “present or past main health problems”, there are several incongruences; this is due to the self-reported nature of information collected.

The frequency of self-reported adverse effects in relation to each botanical is reported in Table 4; the total number of botanical ingredients contained in PFS with reported adverse effects was 72 and the total counts were 144. In most cases (46%), the PFS involved contained one ingredient. Forty botanicals (55.6% of the total) were associated with a single adverse event and 80% of them were included in PFS containing two or more ingredients. Considering the most prevalent botanicals associated with adverse effects, 14 were associated with 68 reported adverse effects, representing the 47.2% of the total counts. In particular, eight were due to *Valeriana officinalis* (seven of them in Spain), seven to *Camellia sinensis*, six to *Ginkgo biloba* and six to *Paullinia cupana*.

The association of adverse effects with different organ systems is listed in Table 5.

**Table 5:** Distribution of adverse effects among the different organ systems.

System	Number of reports	Percentage of total
Gastrointestinal system	52	59.8
Nervous system	15	17.2
Cardiovascular system	4	4.6
Skin and hair	3	3.4
Hepatotoxicity	2	2.3
Urinary tract	2	2.3
Immune system (Allergy)	2	2.3
Other	7	8.0
**Total**	**87**^a^	**100**

^a^ The total number of adverse effects reported is 87 since 5 out of 82 consumers complained about two PFSs

Approximately 60% of adverse events were related to the gastrointestinal tract, distributed between gastric problems (where nausea was the most reported symptom) and intestinal effects (mainly diarrhoea).

The nervous system was the second most affected area with nine cases of insomnia, four of dizziness and two of migraine; the cardiovascular system was reported in four cases of tachycardia. The stimulating effects of botanicals containing caffeine can explain the insomnia and tachycardia reported by consumers 13, 46, 55, and 58, but in other cases, the effects were unexpected. An example is the case of insomnia due to *Valeriana officinalis* (ID 49 and ID 64), which is the opposite of the usual physiological effect, associated with this botanical [11–12]. *Ginkgo biloba* was involved in three cases of insomnia and one of dizziness.

Hair and skin were affected in three cases. A case of hair loss was reported by consumer 10, taking *Equisetum arvense* 3 units/day for 12 months. This adverse effect could be associated with the reported effect of *E*. *arvense* in reducing the bioavailability of thiamine after chronic consumption [50]. On the other hand, the role of thiamine deficiency in hair loss has been hypothesised but insufficiently documented [79]. Hepatotoxicity, defined as an increased level of liver enzymes, was reported by two consumers using red rice (*Oryza sativa* fermented by the fungus *Monascus purpureus*). Red rice is widely used in mild hypercholesterolemia, as a "natural" alternative to statins. Several side effects have been described in consumers using this ingredient, such as headache, dizziness, heartburn, gas and digestive tract discomfort, and it should be used cautiously by people suffering from liver disease and those at risk of it [52–53]. Allergies to *Allium sativum* and to a PFS containing *Melissa officinalis* and *Olea europaea* were reported by two consumers (ID 62 and ID 21, respectively).

A difficulty in swallowing was reported by a consumer (ID 19), using red rice to reduce blood cholesterol. Even though this effect has not been previously associated with red rice, there are some reports concerning the statins (having similar biological activity) for which the impaired swallowing was considered among possible symptoms of muscle degeneration [55].

Table 6 compares the plants most prevalently involved in adverse effects as reported by the PlantLIBRA project, in relation to: 1) data from the literature [9], 2) reports from Poisons Centers [80]; and finally from this study.

**Table 6:** Plants most frequently involved in adverse effects as reported from three sources in the PlantLIBRA project.

Review from literature [9]		Data from Poisons Centers		Self-reported adverse effects (PFS Consumer survey)	
Plant	%^a^	Plant	% ^a^	Plant	%^a^
*Glycine max*	19.3	*Valeriana officinalis*	14.3	*Valeriana officinalis*	9.2
*Glycyrrhiza glabra*	12.2	*Camellia sinensis*	6.2	*Camellia sinensis*	8.0
*Camellia sinensis*	8.7	*Melissa officinalis*	4.3	*Ginkgo biloba*	6.9
*Ginkgo biloba*	8.5	*Mentha x piperita*	4.3	*Paullinia cupana*	6.9
*Citrus aurantium*	5.1	*Passiflora incarnata*	4.3	*Cynara scolymus*	5.7
*Cinnamomum verum*	4.7	*Paullinia cupana*	4.3	*Echinacea spp*.	5.7
*Cimicifuga racemosa*	4.7	*Glycyrrhiza glabra*	3.7	*Olea europaea*	5.7
*Echinacea purpurea*	4.1	*Ilex paraguariensis*	3.7	*Oryza sativa+ Monascus purpureus (Red rice)*	5.7
*Vitex agnus-castus*	3.9	*Panax ginseng*	3.1	*Panax ginseng*	5.7
*Hypericum perforatum*	3.9	*Citrus aurantium*	2.5	*Equisetum arvense*	4.6
*Panax ginseng*	3.3	*Cynara scolymus*	2.5	*Allium sativum*	3.4
*Valeriana officinalis*	2.8	*Dioscorea villosa*	2.5	*Foeniculum vulgare*	3.4
*Vitis vinifera*	2.8	*Allium ursinum*	1.9	*Glycine max*	3.4
**Total cases**	**492**	**Total cases**	**161**	**Total cases**	**87**

^a^number of counts/total cases

It is important to underline that the review from the literature did not separate cases due to botanicals used as food supplements or traditional medicines as was the case in the other two data reviews. Moreover, due to the very high number of botanicals in PFS, the review on the scientific literature included "only" 66 among the most frequently consumed botanicals. The lists of plants most reported by Poisons Centers and by the consumers' survey are similar, sharing five out 13 botanicals; among them *Valeriana officinalis* (garden valerian) and *Camellia sinensis* (tea) were in the first two positions. Moreover, a similar position in the ranking was occupied by *Paullinia cupana* (guarana), *Cynara scolymus* (globe artichoke), and *Panax ginseng* (chinese ginseng).

## Conclusions

The cases of adverse effects described here were self-reported and thus without any supporting clinical evidence; the agreement with data published in scientific papers and in particular with the survey performed by the PlantLIBRA project among Poisons Centers allows the following conclusions:

1. As reported previously, severe adverse effects related to PFS are quite rare [80];
2. Mild or moderate adverse symptoms can be present but most of them do not require clinical support;
3. Data reported in this paper confirm that some plants are more frequently involved in adverse effects than others and can help family doctors, among other health professionals, to become aware about the possible consequences of the increasing use of food supplements containing botanicals;
4. This information could also be used to educate the public as to the possibility of adverse effects associated with the consumption of these food supplements.
